# Two-step deposition of Ag nanowires/Zn_2_SnO_4_ transparent conductive films for antistatic coatings†

**DOI:** 10.1039/d1ra00427a

**Published:** 2021-04-21

**Authors:** Jing Li, Fengmei Cheng, Haidong Li, Hongwen Zhang, Gang Wang, Daocheng Pan

**Affiliations:** School of Materials Science and Engineering, Changzhou University Changzhou Jiangsu 213164 China; College of Material and Textile Engineering, Jiaxing University Jiaxing Zhejiang 314001 China hdlipr@163.com; State Key Laboratory of Rare Earth Resource Utilization, Changchun Institute of Applied Chemistry, Chinese Academy of Sciences 5625 Renmin Street Changchun Jilin 130022 China pan@ciac.ac.cn

## Abstract

Silver nanowire (AgNW) networks play an important role in the transparent conductive electrodes or antistatic coatings. In this work, we describe a facile two-step method to fabricate AgNWs/Zn_2_SnO_4_ composite films. Long AgNWs with a high aspect ratio were prepared through a modified polyol method, in which the organic octylamine hydrochloride rather than the commonly used inorganic chlorides was used as the shape-controlling agent. The AgNW networks were fabricated on the glass substrate, on which the Zn_2_SnO_4_ film was deposited, forming robust AgNWs/Zn_2_SnO_4_ composite films. The as-prepared composite films have strong adhesion, high thermal stability, low sheet resistance (5–15 ohm sq^−1^) and high light transmittance (85–80%), indicating a promising application prospect for transparent conductive electrodes and antistatic coatings.

## Abstract 2

Robust and stable AgNWs/Zn_2_SnO_4_ hybrid thin films with a sheet resistance of 5–15 ohm sq^−1^ and transmittance of 85–80% are fabricated.

## Graphical abstract

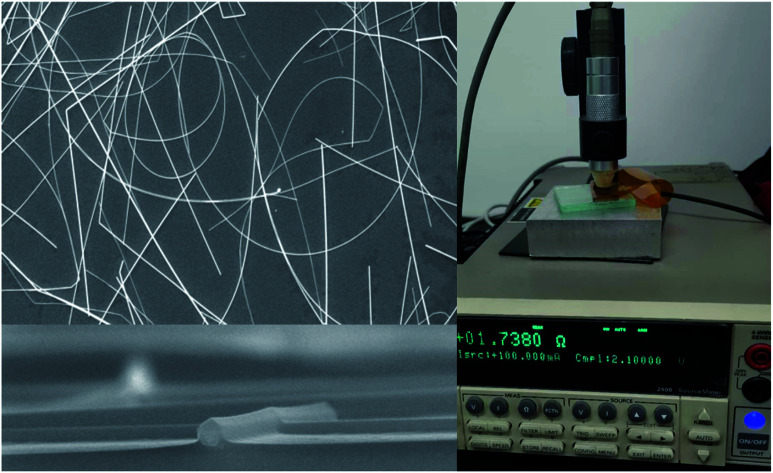

## 1. Introduction

Electrostatic charges can easily accumulate on the surfaces of many insulators, such as glass, ceramics, plastics, rubber and paper, which are harmful on many occasions. For example, static charges often bring about the electrostatic adhesion of dust, and discharging may lead to breakdown of electronic components, and in some situations can even cause combustion or an explosion.^1–3^ To solve the issue of charge accumulation, some conductive materials, such as 2-D layered MXENE,^4^ conductive polymers,^5–10^ conductive oxides,^11–16^ carbon materials,^17–19^ and metal nanowires,^20–28^ are adopted as the antistatic materials to be coated on the surface of some insulating materials. Among these materials, the conductive polymers have relatively poor conductivity and stability, thus they are not suitable for actual applications especially in harsh space environmental conditions.^29^ Conductive oxides such as indium–tin-oxide (ITO),^30–32^ fluorine-doped SnO_2_ (FTO),^33,34^ antimony-doped SnO_2_ (ATO)^35–37^ and aluminium-doped ZnO (AZO)^38,39^ have good stability and conductivity, but their applications are hindered by their vacuum-based preparation methods. The magnetron sputtering method cannot meet the requirements of new flexible and stretchable electronic products and large-area spacecrafts for antistatic coatings.^12,13^ Graphene and carbon nanotubes are good antistatic materials, but their preparation costs are high and their conductivity is relatively poor.^17–19^ By contrast, metal copper and silver nanowires (AgNW), especially the latter, are of higher electrical conductivity, promising thermal stability, and low preparation cost, become the ideal candidate for the antistatic coating materials.

Like the conductive oxides, the conductive AgNW networks are of high light transmittance, which widen their application as the conducting layer for antistatic coatings and even as the transparent and conductive electrodes for optoelectronic devices.^20–25^ Unlike the conductive oxides, the adhesion between AgNWs and the substrate is poor, thus an inorganic or organic matrix is needed to form a composite film with AgNWs. The introduction of the matrix not only improves the adhesive force of the AgNWs, but also increases the stability of AgNWs and the electroconductivity of the antistatic layer. For instances, coated with TiO_2_ nanoparticle film, the stability of AgNWs against oxygen is greatly improved since the oxygen is isolated outside. The electron conductivity of TiO_2_ helps to collect and transport the electron between the substrate and the AgNW network, namely, to improve the short-range conductivity.^40^ In addition to TiO_2_, however, only a few oxides such as ZnO,^41^ MoO_3_,^42,43^ WO_3_,^44^ SnO_2_ (ref. 45 and 46) and AZO^47,48^ have been adopted as the inorganic matrix for AgNWs, and more inorganic matrices still need to be developed. Herein, we demonstrate a new inorganic matrix, *i.e.* Zn_2_SnO_4_, as the matrix for AgNWs networks. In this work, long AgNWs with a high aspect ratio were prepared through a modified polyol method, in which the organic octylamine hydrochloride rather than the common inorganic chlorides was used as the shape-controlling agent. The conductive networks were prepared by spraying our homemade AgNWs on glass substrates. On the AgNW networks, the Zn_2_SnO_4_ film was deposited by spin-coating a Zn–Sn–O precursor solution, followed by annealing at 200 °C. The as-prepared AgNWs/Zn_2_SnO_4_ composite films showed high adhesion, good conductivity, high transmittance and excellent thermal stability, indicating that these AgNWs/Zn_2_SnO_4_ composite films have promising prospects in the field of antistatic coatings.

## 2. Experimental section

### 2.1 Materials

Polyvinylpyrrolidone (PVP, MW = 360 000), zinc acetate (99.9%), tin dichloride (99%), *n*-octylamine (99%), *n*-butylamine (99%), *n*-butyric acid (99%), ethylene glycol (EG, AR) were purchased from Aladdin Inc. Silver nitrate (AgNO_3_, AR) was bought from Sinopharm Chemical Reagent Co., Ltd. Ethanol and hydrochloric acid were purchased from Xilong Scientific Co., Ltd. All chemicals were used without further purification. Octylamine hydrochloride was synthesized by the reaction of *n*-octylamine with hydrochloric acid in ethanol.

### 2.2 Preparation of AgNWs

First, two stock solutions were prepared: (A) 2.5 mM octylamine hydrochloride, (B) 200 mM AgNO_3_ in ethylene glycol. Next, 0.4 g PVP and 40 mL of EG were loaded into a 100 mL of flask. The solution was stirred at 100 °C, and 2 mL of the solution A and 10 mL of the solution B were added into the flask to form a homogeneous solution. The solution was poured into a 100 mL of Teflon liner of a stainless autoclave and was put in a pre-heated oven (115 °C) and was incubated for 11 h. After that, the autoclave was took out and cooled to the room temperature. The as-prepared AgNWs were purified by filtering and then were collected and redispersed in ethanol.

### 2.3 Preparation of the Zn_2_SnO_4_ precursor solution

0.2752 g zinc acetate was dissolved in a mixture of 0.5 mL of *n*-butyric acid, 0.5 mL of *n*-butylamine and 10 mL of ethanol by magnetic stirring and heating at 100 °C, then the solution was cooled to the room temperature. After that, 0.1422 g tin dichloride was added to form a mixed Zn–Sn–O precursor solution. The as-prepared Zn_2_SnO_4_ precursor solution was stored for future use.

### 2.4 Fabrication of AgNWs/Zn_2_SnO_4_ composite films

The glass substrates were washed in deionized water and ethanol for 30 min to remove the dust and impurities, and were dried by the nitrogen flow. The AgNW dispersion was spray-coated onto the glass substrate by a spray gun. The spray distance and pressure were 8.0 cm and 150 kPa, respectively. The density of nanowires in the AgNWs network was controlled by the spray time. Finally, Zn_2_SnO_4_ precursor solution was spin-coated (2600 rpm, 20 s) onto the AgNW networks and then was annealed at 200 °C for 5 min to form the AgNWs/Zn_2_SnO_4_ composite film. Note that the thickness of Zn_2_SnO_4_ thin film can be changed by adjusting spin-coating rate or tuning the concentration of Zn_2_SnO_4_ precursor solution (see Table S1 in ESI†), and the thickness of Zn_2_SnO_4_ layer in the AgNWs/Zn_2_SnO_4_ composite films used for all characterizations is about 24 nm only if otherwise pointed out.

### 2.5 Characterizations

The UV-vis transmission spectra were collected on a Metash UV-5200 spectrophotometer, recorded from 400–1000 nm. The X-ray diffraction (XRD) pattern was measured on a Bruker D8 Focus diffractometer equipped with a Cu Kα radiation (*λ* = 0.15405 nm) source. The SEM images were acquired on a Hitachi-4800 field-emission scanning electron microscope (SEM, Hitachi, Ltd., Japan), operated at an accelerating voltage of 10 kV. The optical images were captured on a Phenix HD digital camera (MC-D800U). The sheet resistance was measured by the four-point probe method with a Keithley 2400 source meter (sheet resistance = measured resistance × 4.532). Variable temperature experiment was carried out on a ET9000 electric transport measurement instrument (East Changing Inc., China) equipped with a 280 temperature controller and a Cryodyne refrigerator (CTI-Cryogenics Helix Technology Corporation, USA), accompanied by the transmission measurement at 550 nm on a Metash UV-5200 spectrophotometer.

## 3. Results and discussion

**Fig. 1:**
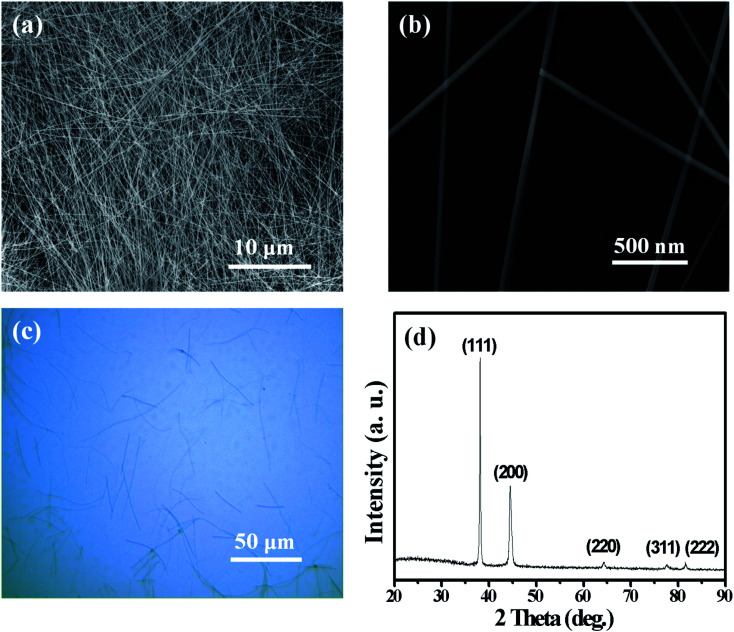
SEM images of our home-made AgNWs at low (a) and high (b) magnifications; the optical microscope image (c) and XRD pattern (d) of AgNWs.

Silver nanowires of high purity were prepared by our modified polyol method, in which organic octylamine hydrochloride, instead of commonly used inorganic chlorides, was adopted as the shape-controling agent. Fig. 1a–c show the morphologies of as-prepared AgNWs, indicating that these AgNWs have an average diameter of ∼130 nm and an average length of ∼95 μm. Long AgNWs with high aspect ratios is beneficial for lowering the sheet resistance and improving the transparency of the AgNW networks. The XRD pattern in Fig. 1d, including the peak positions and the intensity ratio of these peaks are consistent with those described in literature,^49^ indicating that these AgNWs may have a pentagonally twinned structure, as is confirmed in Fig. 3c.

**Fig. 2:**
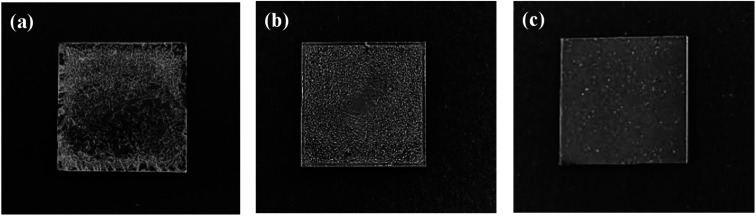
Photographs of AgNWs films prepared by three different methods: (a) drop-casting method; (b) spin-coating method; (c) spray-coating method.

**Fig. 3:**
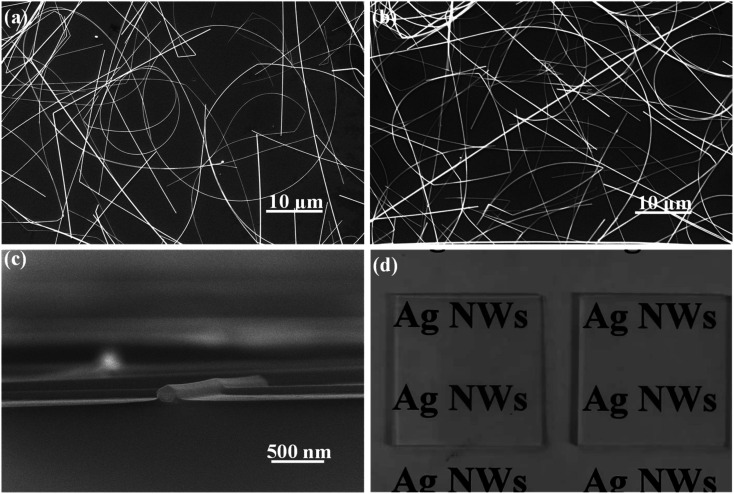
SEM images of pristine a AgNW network (a) and a AgNWs/Zn_2_SnO_4_ composite film (b); (c) cross-sectional SEM image of the AgNWs/Zn_2_SnO_4_ composite film; (d) photographs of a pristine AgNW network/glass sample (left) and a AgNWs/Zn_2_SnO_4_/glass sample after a tape peeling test (right).

In order to fabricate better AgNW networks, three types of solution-based deposition methods, namely drop-casting, spin-coating and spray-coating, have been tried. As shown in Fig. 2a, the AgNWs cannot be uniformly distributed on the glass substrate using the drop-casting method, causing a inhomogeneous resistance distribution in the conducting film. We also did not get well distributed AgNW networks by the spin-coating method (see Fig. 2b). When the concentration of AgNWs ink is low, it is difficult to spin-coat AgNWs on the glass substrate due to centrifugal action, and the coffee ring may form,^50^ which will severely affect the conductivity of the network. If the concentration of AgNWs ink is high, the conductivity of the film will be good, but the light transmittance will be poor. Moreover, the AgNWs trend to agglomerate during the spin-coating process, resulting in a rough surface of the thin film and a high resistance. Compared with the above two methods, the spray-coating, a well-known solution processing method that widely used in industrial laboratories for film preparation, is a more effective way for distributing AgNWs, especially on some irregular surfaces.^51^ As Fig. 2c shows, the glass substrate with the AgNW networks prepared by a spray-coating method has a relatively smooth surface. It was observed that the AgNWs are uniformly distributed on the glass and overlaped each other (Fig. 3a), forming a conductive network. More importantly, the density of AgNWs in the network can be tuned simply by altering the spray time, accompanied by a simultaneous regulation of the resistance and transmission of the network.

**Fig. 4:**
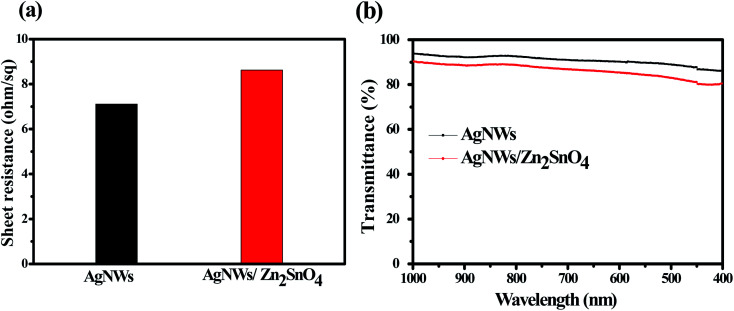
The sheet resistance (a) and light transmission spectra (b) of the AgNWs film and the AgNWs/Zn_2_SnO_4_ composite film.

Generally, the as-prepared pristine AgNW networks cannot be used directly as the antistatic coating due to their weak adhesion with the substrate. The AgNWs are usually added into an inorganic or organic matrix first and then are casted onto the substrate to form a composite film, which can significantly improve the adhesion between the AgNW networks and the substrate. Recently, a few inorganic oxides such as TiO_2_, ZnO, MoO_3_, WO_3_, SnO_2_ and AZO have been investigated for fixation of AgNWs on some substrates. Herein, we use a new inorganic matrix, Zn_2_SnO_4_, to anchor AgNW networks on glass substrate. Different to the routine one-step processing method, the preparation of AgNW/Zn_2_SnO_4_ herein is divided into two steps, *i.e.* spray-coating the AgNWs first and then spin-coating the Zn_2_SnO_4_ layer. The upper Zn_2_SnO_4_ layer was prepared by spin-coating the mixed Zn–Sn–O precursor solution, followed by a low-temperature sintering process at 200 °C for 5 min. During sintering, zinc and tin salts hydrolyze to form a fine, smooth and uniform transparent Zn_2_SnO_4_ thin film (see Fig. S1†), covering the AgNW networks. Owing to the low sintering temperature, the as-prepared Zn_2_SnO_4_ semiconductor film possesses an amorphous structure and contains lots of oxygen vacancy defects,^52^ which has a high electron mobility of Zn_2_SnO_4_,^53^ helping to transfer electrons between the glass substrate and the AgNW networks. XRD patterns of as-prepared AgNW/Zn_2_SnO_4_ composite thin film and the corresponding thin film annealed at 450 °C for 30 min are shown in Fig. S2.† It was found that Zn_2_SnO_4_ matrix thin film annealed at 450 °C for 30 min is amorphous. If pristine Zn_2_SnO_4_ thin film were annealed at 600 °C for 1 h in air, XRD characteristic peaks for cubic Zn_2_SnO_4_ can be clearly observed in Fig. S3,† which is consistent with the literature report.^54^ Note that XRD patterns of ZnO or SnO_2_ impurities are not observed. To further confirm homogenous composition for as-prepared AgNW/Zn_2_SnO_4_ composite film, we measured SEM image and the corresponding elemental maps for as-prepared AgNW/Zn_2_SnO_4_ composite film, as shown in Fig. S4.† It was clearly observed that Zn and Sn elements have a homogeneous distribution, indicating that ZnO or SnO_2_ impurities should not exist. The sheet resistance showed a neglectable increase from 7.11 ohm sq^−1^ to 8.62 ohm sq^−1^ after the deposition Zn_2_SnO_4_ (see Fig. 4a). No obvious alteration in AgNW distribution is observed after deposition of the upper Zn_2_SnO_4_ layer (Fig. 3a and b), except that the AgNWs are closely anchored on the glass surface by an ultra-thin Zn_2_SnO_4_ layer (Fig. 3c), forming a firm AgNWs/Zn_2_SnO_4_ composite films. Fig. 3d shows the optical picture of AgNW networks before and after Zn_2_SnO_4_ deposition, indicating a neglectable decline of transmission (from 91.48% to 89.73%) for the visible light (see Fig. 4b). It should be pointed out that the right one in Fig. 3d is a sample which had passed by a tape-peeling test and its conductivity remained unchanged. No discernible damage was observed for the AgNWs/Zn_2_SnO_4_ composite film, whereas the AgNW networks on the left sample can be easily peeled off from the glass substrate and leaves the AgNW networks on the tape, revealing a significant improvement of adhesion after Zn_2_SnO_4_ deposition.

**Fig. 5:**
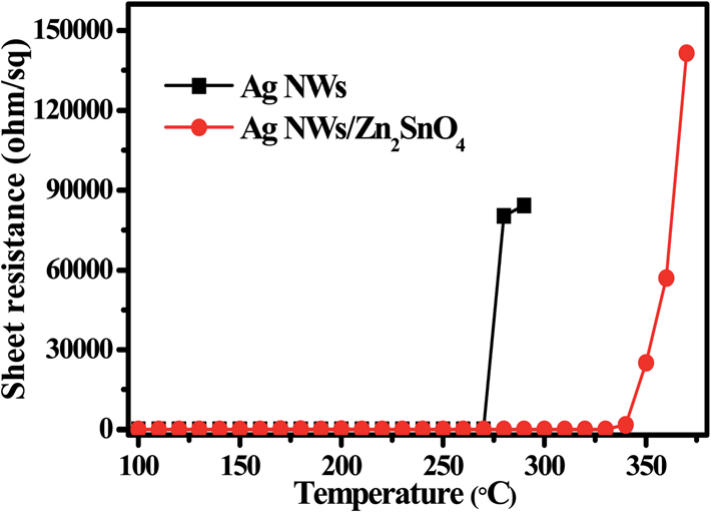
Sheet resistance of a bare AgNW network and a AgNWs/Zn_2_SnO_4_ composite film heated at different heating temperatures.

**Fig. 6:**
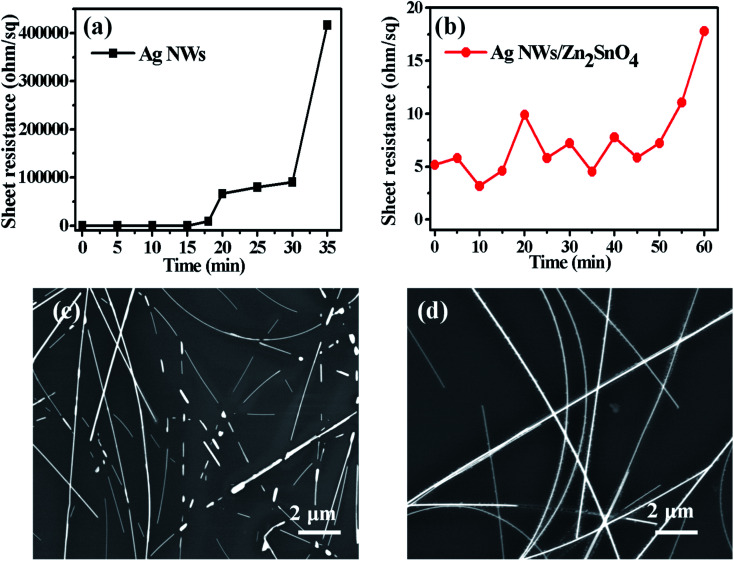
The thermal stability tests for a pristine AgNW network (a) and a AgNWs/Zn_2_SnO_4_ composite film (b) heated at 275 °C for different time. The SEM images of a pristine AgNW network (c) and a AgNWs/Zn_2_SnO_4_ composite film (d) heated at 275 °C for 18 and 60 min, respectively.

**Fig. 7:**
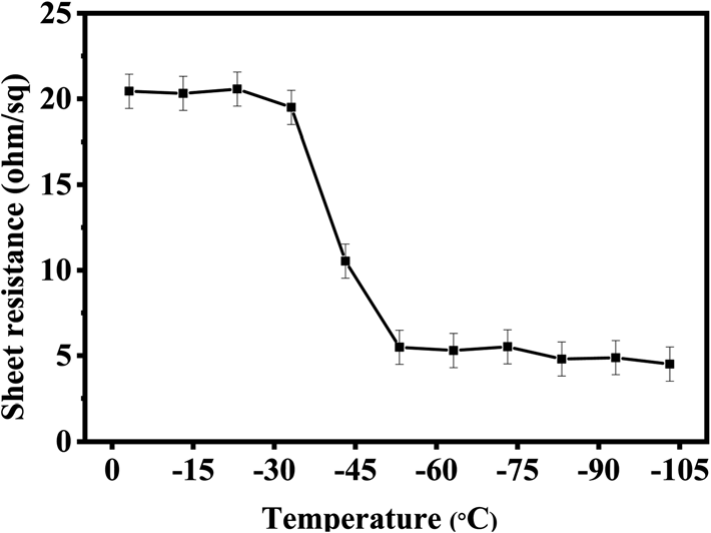
The variation of sheet resistance of a AgNWs/Zn_2_SnO_4_ composite film at low temperatures.

Thermal test was also carried out to investigate the stability of AgNW networks and AgNWs/Zn_2_SnO_4_ composites, which is of high importance for their future application. As shown in Fig. 5, both pristine AgNW networks and AgNWs/Zn_2_SnO_4_ composite films are stable below 280 °C when the thermal treatment is set at 10 min. Above this point, the pristine AgNW networks begins to lose their conductivity (totally at 300 °C), whereas the AgNWs/Zn_2_SnO_4_ composite film is still stable until the temperature reaches 330 °C and totally lose its conductivity at 380 °C. On the other hand, if the temperature is set at 275 °C, the pristine AgNW networks are stable in 15 min, and then begin to lose their conductivity. As shown in Fig. 6c, the rising of the sheet resistance is attributed to the partial melting of some silver nanowires, leaving disconnected silver nanorods or nanoparticles. When the thermal treatment comes to 35 min, the AgNW networks almost lose their conductivity, indicating fusion of most AgNWs in the networks. By contrast, the AgNWs in AgNWs/Zn_2_SnO_4_ composite did not show distinct variation in morphology in 50 min (Fig. 6b), showing higher thermal stability than that of the pristine AgNW networks. Above this point of time, however, very small silver nanoparticles start to form around the nanowires (Fig. 6d), shrink slowly and disappear in the end, accompanied by a loss in conductivity. The electrical conductivity of a AgNWs/Zn_2_SnO_4_ composite film at low temperatures was also investigated. As shown in Fig. 7, the resistance of the AgNWs/Zn_2_SnO_4_ composite film is stable above −23 °C, and then drop dramatically (20.44 ohm sq^−1^ to 4.51 ohm sq^−1^) at about −33 °C until it reaches a platform again at about −53 °C. This unnormal curve may result from our test sets, test fixtures, poor thermal conductivity of glass substrate and two-probe based test method herein. Anyway, the low-temperature test proves that the AgNWs/Zn_2_SnO_4_ composite film has a better electrical conductivity at low temperatures. Based on these variable temperature experiments, it can be concluded that our AgNWs/Zn_2_SnO_4_ composite film has a wide usable temperature range of −103 to 330 °C, which can meet the requirements of thermal stability for antistatic coatings in various fields. In addition, the stability of AgNWs/Zn_2_SnO_4_ composite film against ultraviolet light illumination could be improved by using a previously reported method.^55^

## 4. Conclusions

In summary, long silver nanowires with high aspect ratios were prepared by a modified polyol method using organic octylamine hydrochloride as the shape-controling agent. Transparent and conductive AgNWs/Zn_2_SnO_4_ composite films were fabricated by employing a two-step (spray-coating AgNWs and spin-coating Zn_2_SnO_4_) method on the glass substrate, which exhibited high electric conductivity (5–15 ohm sq^−1^) and light transmittance (85–80%). The thermal stability and adhesion of AgNWs/Zn_2_SnO_4_ composite films were dramatically improved by deposition of Zn_2_SnO_4_ matrix. The as-prepared transparent conductive AgNWs/Zn_2_SnO_4_ composite films can be a good substitute for the commercial transparent conductive metal oxides, such as ITO, FTO, and AZO. More importantly, these robust and stable AgNWs/Zn_2_SnO_4_ films have a wide usage temperature range of −103 to 330 °C, indicating a promising application prospect for antistatic coatings.

## Conflicts of interest

There are no conflicts to declare.

## Supplementary Material

RA-011-D1RA00427A-s001
