# Supplementary material for: Two-step deposition of Ag nanowires/Zn2SnO4 transparent conductive films for antistatic coatings
Source: RSC Adv. 2021 Apr 21;11(24):14730–6. doi: 10.1039/d1ra00427a (PMC8697850; doi:10.1039/d1ra00427a)
Supplement: RA-011-D1RA00427A-s001 [file RA-011-D1RA00427A-s001.pdf]

Electronic Supplementary Material (ESI) for RSC Advances.
This journal is © The Royal Society of Chemistry 2021

Two-step deposition of Ag nanowires/Zn_2SnO_4 transparent conductive electrodes for antistatic coatings

Jing Li[a, b], Fengmei Chen[b], Haidong Li[b*], Hongwen Zhang[a], Gang Wang[c] and Daocheng Pan[c*]

[a]*School of Materials Science and Engineering, Changzhou University, Changzhou, Jiangsu 213164, China;*

[b]*College of Material and Textile Engineering, Jiaxing University, Jiaxing, Zhejiang 314001, China;*

E-mail: hdlipr@163.com

[c]*State Key Laboratory of Rare Earth Resource Utilization, Changchun Institute of Applied Chemistry, Chinese Academy of Sciences, 5625 Renmin Street, Changchun, Jilin 130022, China*

E-mail: pan@ciac.ac.cn

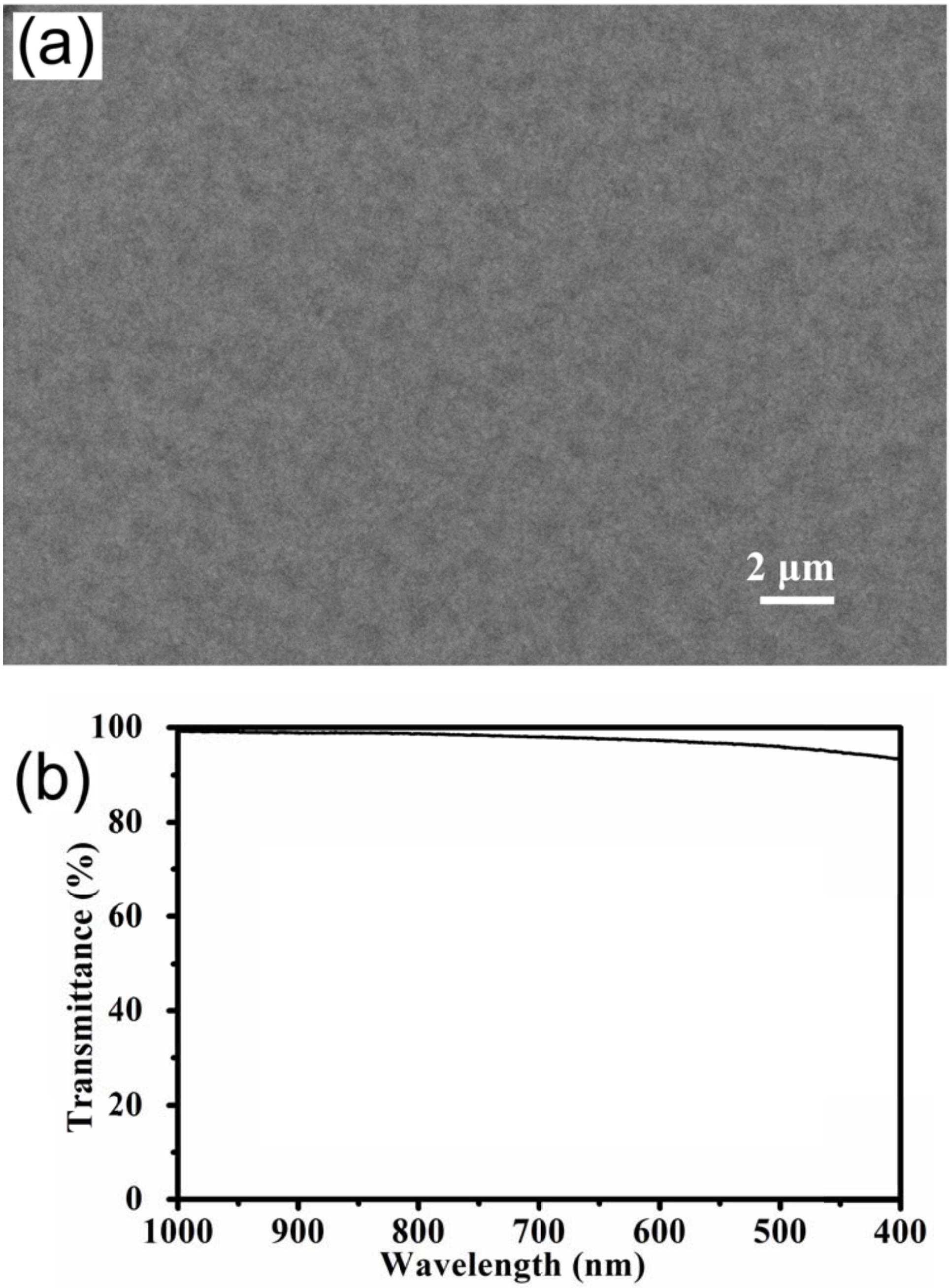

Fig. S1. SEM image (a) and transmission spectrum (b) of a pure Zn_2SnO_4 film.

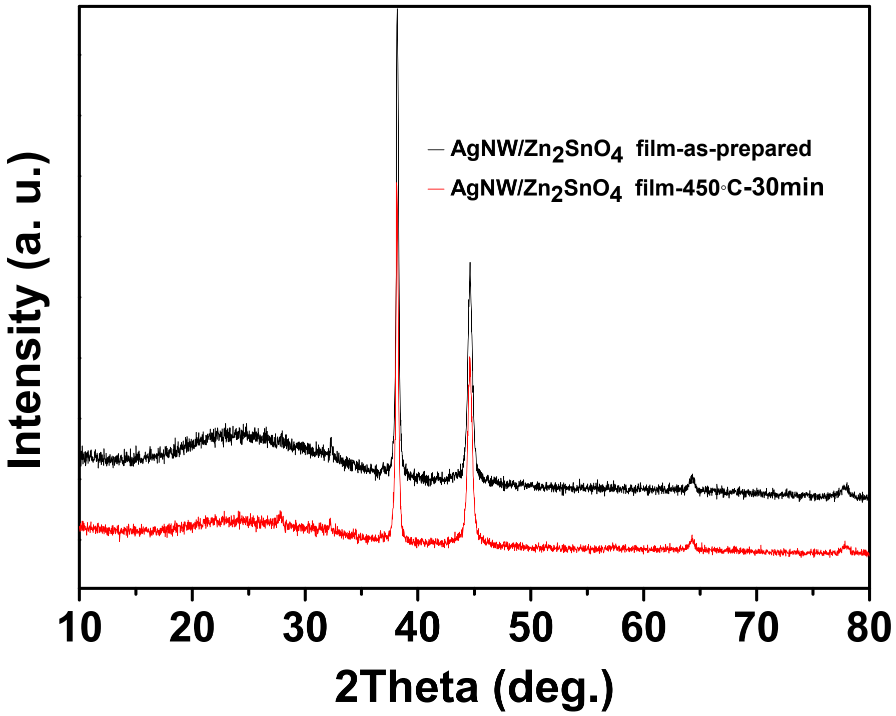

Fig. S2. XRD patterns of as-prepared AgNW/Zn_2SnO_4 composite thin film and the corresponding thin film annealed at 450°C for 30min.

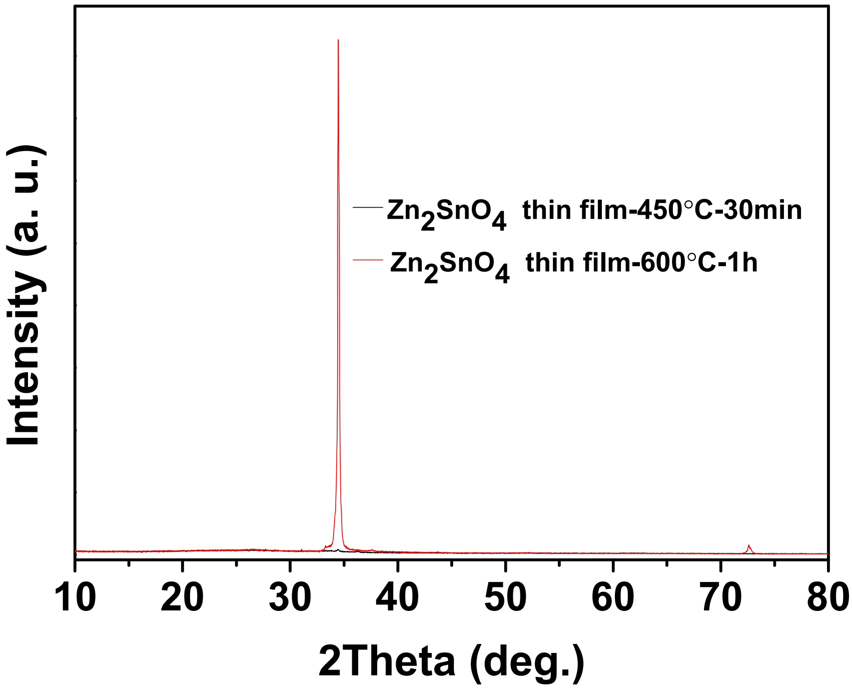

Fig. S3. XRD patterns of pristine Zn_2SnO_4 thin films (~1.8 μm) annealed at 450 and 600 ^{o}C, respectively.

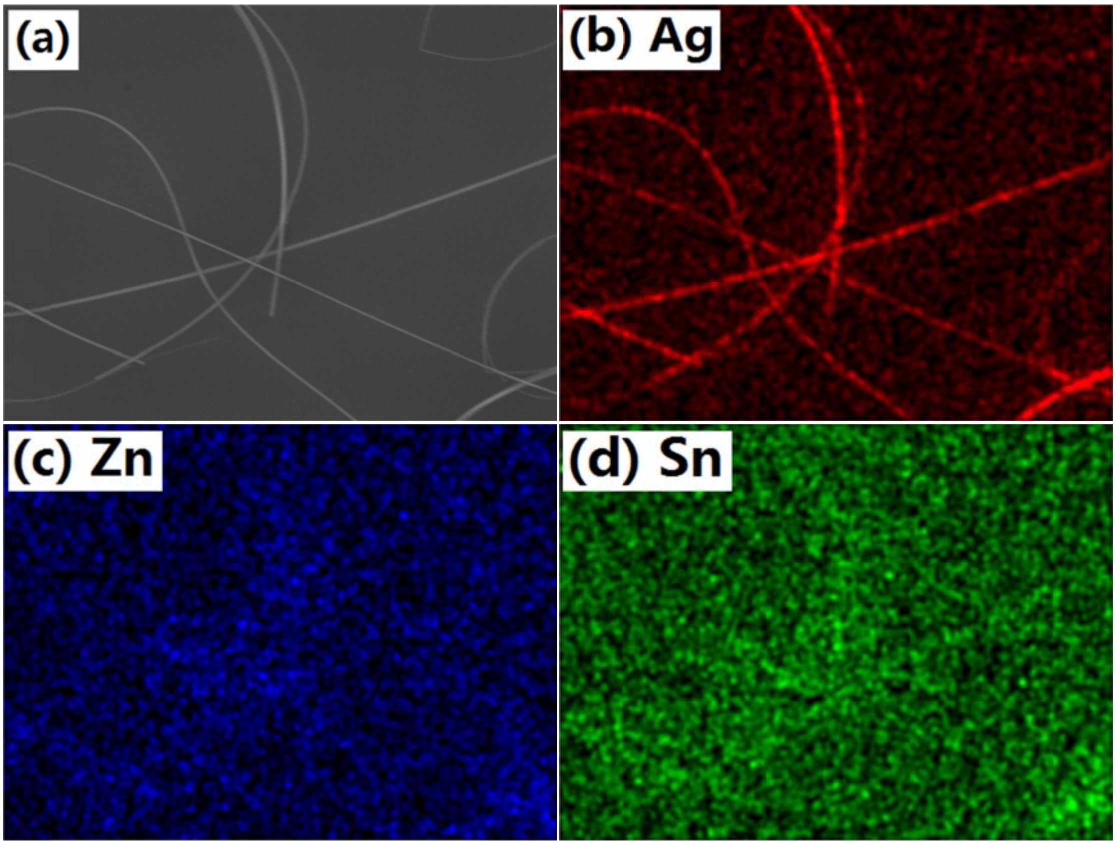

Fig. S4. SEM image (a) and corresponding elemental maps (b, c and d) of as-prepared AgNW/Zn_2SnO_4 composite thin film on silicon wafers.

Table S1. The relationships between the thickness of the Zn_2SnO_4 layer and the concentration of Zn_2SnO_4 precursor solution as well as the spin-coating rate.

Thickness of the Zn_2SnO_4 layer (nm)				
	1 mL ethanol	2.5 mL ethanol	5 mL ethanol	10 mL ethanol
2000 rpm, 10 s	855	335	156	56
2600 rpm,10 s	770	293	120	51
2600 rpm, 20 s	629	250	64	24
